# A Case of Epidural Hematoma after Lumbar Spine Surgery in a Hemophilia B Carrier

**DOI:** 10.31662/jmaj.2024-0005

**Published:** 2024-08-23

**Authors:** Yushi Sakamoto, Tomonori Ozaki, Shogo Tahata, Toru Fujimoto, Seiichiro Naruo

**Affiliations:** 1 Department of Spine Surgery, Naruo Orthopedic Hospital, Kumamoto, Japan

**Keywords:** hemophilia B carrier, spinal canal stenosis, postoperative epidural hematoma, spinal surgery, gelatin-thrombin matrix sealants, factor IX replacement

## Abstract

Hemophilia B is a quantitative or qualitative factor IX anomaly that manifests as an X-linked recessive inheritance pattern in which females are carriers. Postoperative epidural hematoma emerges as a typical complication in spinal surgery, although its incidence is infrequent. No documentation of postoperative epidural hematoma in carriers of hemophilia B exists. A 64-year-old female patient presented with progressive pain and muscle weakness in both lower limbs. Despite a history of childbirth and prior colorectal cancer surgery, the patient displayed no abnormal bleeding tendencies. Subsequently undergoing decompression surgery for lumbar spinal canal stenosis, the patient experienced paralysis and pain in both legs within 5 hours postoperatively. A magnetic resonance imaging scan revealed severe spinal canal compression attributed to a postoperative epidural hematoma, prompting emergency decompression surgery that ameliorated symptoms. The application of gelatin-thrombin matrix sealants (GTMS) facilitated hematoma removal, resulting in an uneventful recovery. In a postoperative interview, it was revealed that her grandson was undergoing treatment for hemophilia B. Additionally, she exhibited diminished factor IX levels and was diagnosed as a hemophilia B carrier. A definitive preoperative diagnosis of the carrier status is imperative. In instances where surgical intervention is warranted, the implementation of factor IX replacement and intraoperative hemostasis with GTMS is promising for potentially averting the onset of postoperative epidural hematoma.

## Introduction

Hemophilia B is a congenital coagulation disorder characterized by quantitative or qualitative aberrations in factor IX, following an X-linked recessive inheritance pattern. Typically, males are afflicted, whereas females serve as carriers. Many carriers have reported experiencing abnormal bleeding, presenting symptoms akin to those observed in mild hemophilia in males or specific manifestations such as menorrhagia in females ^(1), (2), (3)^. Postoperative epidural hematoma is a customary complication of spinal surgery, with a reported frequency of approximately 0.1%-0.2% ^(4), (5)^. However, no documented instances of postoperative epidural hematoma in hemophilia carriers exist. This study reports the case of a hemophilia B carrier diagnosed with epidural hematoma following lumbar spinal stenosis surgery.

## Case Report

**Figure 1.**
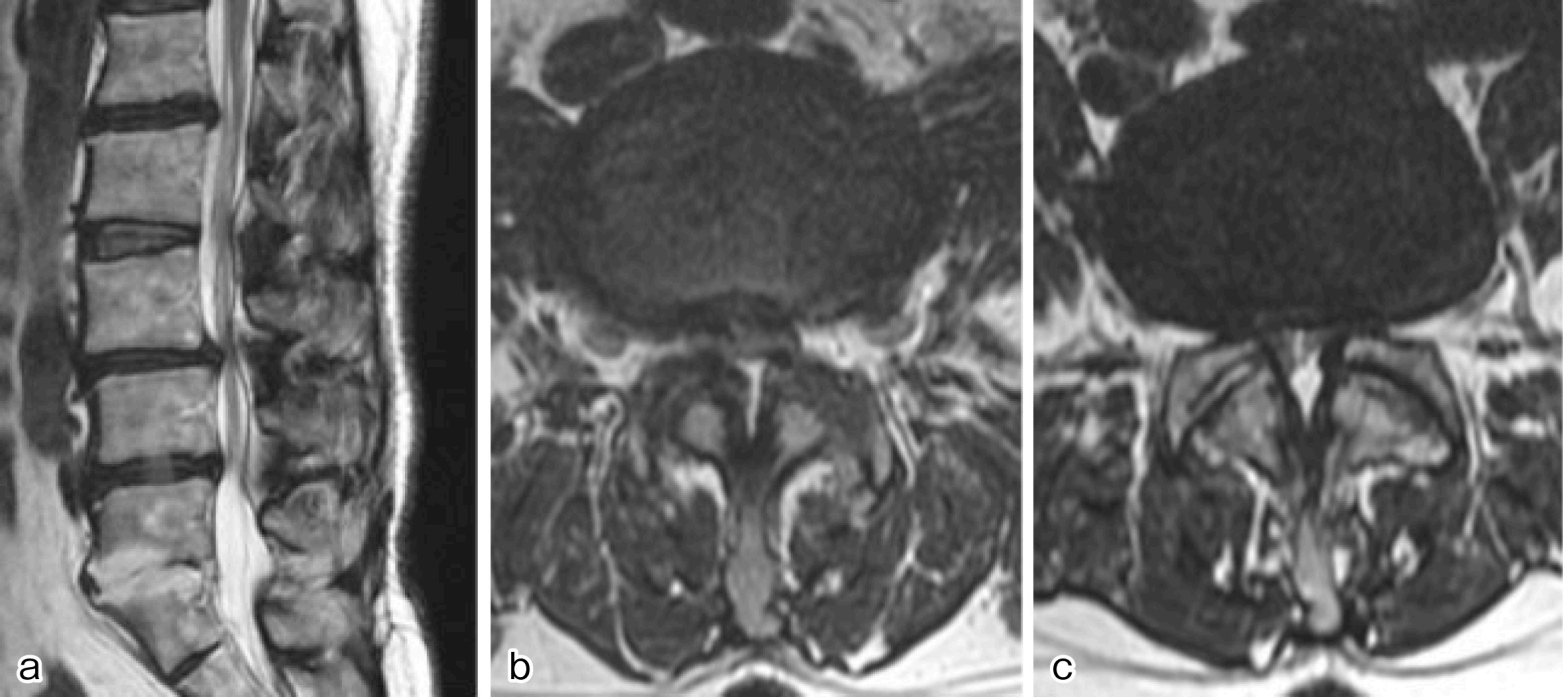
Preoperative magnetic resonance imaging T2-weighted images revealed severe spinal canal stenosis at L3-5. (a) Sagittal section, (b) horizontal section at L3-4, and (c) horizontal section at L4-5.

A 64-year-old woman with a history of uneventful childbirth and colorectal cancer surgery presented with progressively worsening pain in both buttocks and thighs for over 2 years. Bilateral leg weakness ensued, impairing ambulation. Magnetic resonance imaging (MRI) revealed severe spinal canal stenosis due to degenerative lumbar spondylosis at L3-4 and L4-5 (Figure 1), prompting posterior decompression. The preoperative platelet count was 223 × 10^3^/μL, and the activated partial thromboplastin time (APTT) was 31.3 s. Posterior decompression at L3-5 was performed, with an intraoperative blood loss of 80 mL and no abnormal bleeding.

**Figure 2.**
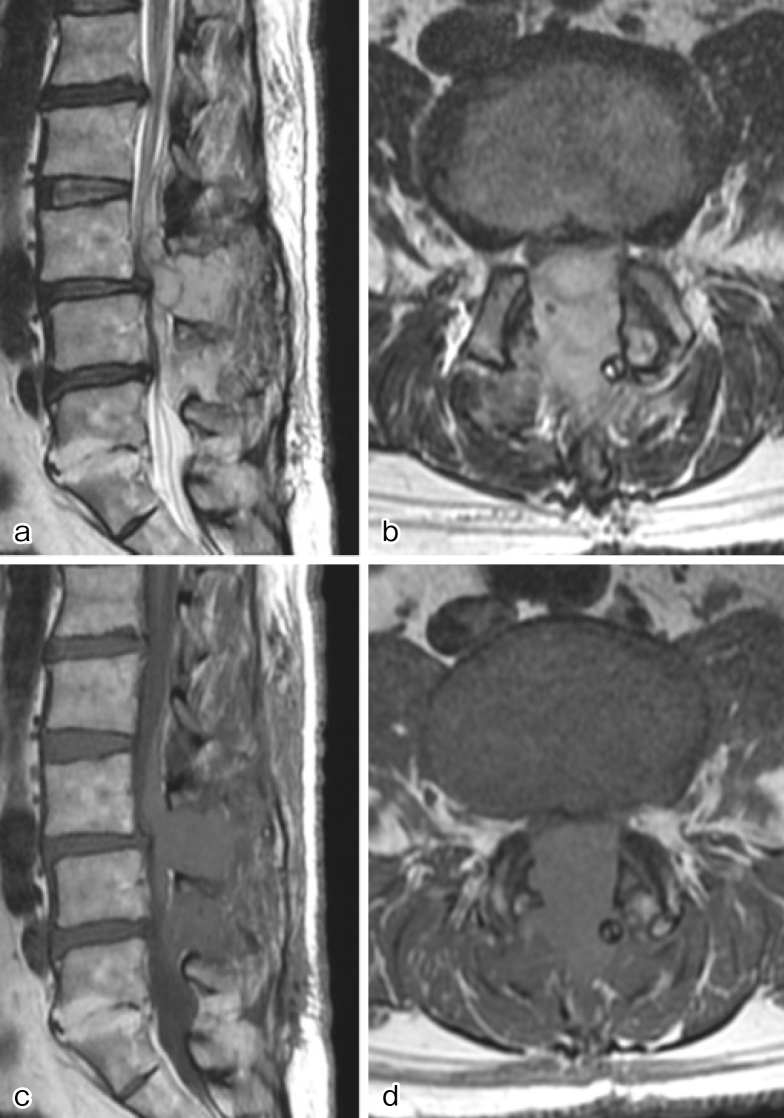
Several hours after the initial surgery, magnetic resonance imaging (MRI) was performed to investigate the cause of bilateral lower limb paralysis and pain. MRI revealed the presence of an epidural hematoma. (a) Sagittal and (b) horizontal sections of T2-weighted images, and (c) sagittal and (d) horizontal sections of T1-weighted images.

The patient manifested ankle paralysis, intense lower back pain, and significant pain in both lower limbs 5 hours postoperatively. At this point, the drainage output was 100 mL. Emergency MRI revealed hematoma formation at the surgical site and severe spinal canal compression (Figure 2). Urgent hematoma removal surgery was performed with no identified source of bleeding. Recognizing persistent bleeding from the epidural venous plexus and muscles, gelatin thrombin matrix sealants (GTMS) facilitated hemostasis. Lower limb pain significantly improved postoperatively, enabling ambulation within 4 weeks and subsequent discharge. Postoperative interviews revealed that the patient’s grandchild is undergoing treatment for hemophilia B. Factor IX measurement indicated a decrease to 45%, and the patient was diagnosed as a hemophilia B carrier.

## Discussion

Hemophilia B, a congenital quantitative or qualitative factor IX disorder, features an X-linked recessive inheritance pattern that predominantly affects males. Female carriers of the condition may exhibit symptoms akin to mild hemophilia in affected males or specific manifestations such as menorrhagia ^(1), (2), (3)^. Patients with hemophilia are categorized as mild if their coagulation factor levels range between 5% and 40%, and in this case, the patient’s level was 45%, surpassing the criteria for mild disease. Consequently, preoperative APTT was within the normal range, and no serious issues were present during prior childbirth or colorectal cancer surgery, making it unfeasible to explore carrier status preoperatively. It is hypothesized that postoperative epidural hematoma could have been averted if factor IX replacement had been performed ^(6)^. In the absence of noticeable abnormal bleeding during the surgical procedure and minimal fluid drainage from the drain, an epidural hematoma may have developed. Therefore, it is imperative to closely monitor the evolution of neurological symptoms following surgical intervention.

The incidence of postoperative epidural hematoma was low at 0.1%-0.22% ^(4), (5)^. and although the initial surgery was unremarkable, paraplegia due to epidural hematoma developed several hours postoperatively. Although no documented cases of postoperative epidural hematoma exist specifically in lumbar spine surgery among hemophilia carriers, speculation indicates that the incidence may surpass that of the general population due to the challenge of relieving pressure when a hematoma forms around the spinal canal and the continuous enlargement of the hematoma due to the inability to achieve hemostasis caused by coagulation abnormalities.

In the second surgery, hemostasis was effectively achieved using GTMS for bleeding from the epidural venous layer, resulting in a favorable outcome. GTMS facilitates hemostasis by converting the patient’s fibrinogen into fibrin and physically controlling blood flow upon contact with blood ^(7), (8)^. Consequently, it is inferred that GTMS contributed to a positive outcome by aiding hemostasis in patients with diminished factor IX levels and coagulation abnormalities.

This case report describes a patient with epidural hematoma following lumbar spine surgery, revealing an undiagnosed hemophilia B carrier status. Knowledge of carrier status before surgery could have averted epidural hematoma through coagulation factor supplementation. Additionally, the application of GTMS for bleeding cessation is considered beneficial for achieving hemostasis in patients with coagulopathy.

## Article Information

### Conflicts of Interest

None

### Author Contributions

Yushi Sakamoto and Tomonori Ozaki: care of patients and manuscript writing, design, and editing. Shogo Tahata: care of patients and manuscript editing. Toru Fujimoto and Seiichiro Naruo: providing guidance for manuscript writing.

### Informed Consent

The patient understood and consented to the anonymous submission of the case report to a medical journal.

### Approval by Institutional Review Board (IRB)

Not applicable.
